# High Rate of Chronic Villitis in Placentas of Pregnancies Complicated by Influenza A/H1N1 Infection

**DOI:** 10.1155/2014/768380

**Published:** 2014-02-16

**Authors:** Wouter J. Meijer, Annemarie M. J. Wensing, Hein W. Bruinse, Peter G. J. Nikkels

**Affiliations:** ^1^Perinatal Center, Wilhelmina Children's Hospital, University Medical Center Utrecht, 3584 CX Utrecht, The Netherlands; ^2^Department of Virology and Medical Microbiology, University Medical Center Utrecht, 3584 CX Utrecht, The Netherlands; ^3^Department of Pathology, University Medical Center Utrecht, 3584 CX Utrecht, The Netherlands

## Abstract

*Introduction*. Pandemic influenza A/H1N1 infection during pregnancy has a negative impact on several aspects of pregnancy outcome. As yet, no elucidating mechanism has been revealed for these effects. We investigated whether placentas of pregnancies complicated by 2009 influenza A/H1N1 infection demonstrated an increased rate of chronic villitis and whether this villitis was caused by influenza virus. *Methods*. We performed a cohort study on 145 pregnant outpatients during the 2009-2010 influenza A H1N1 pandemic. The placentas of patients with influenza infection were examined for histologic signs of chronic villitis. In case of villitis, polymerase chain reaction (PCR) on influenza virus was performed on placental tissue. *Results*. 29 patients had influenza infection. Placentas of 15 of these patients were collected and examined. In 7 cases (47%) chronic villitis was detected. Placental weight and birth weight of the neonates did not differ between cases with and without chronic villitis. In all cases PCR was negative for influenza. *Conclusion*. In our series, chronic villitis was present in a high proportion of placentas of pregnancies complicated by 2009 influenza A/H1N1 infection. We could not demonstrate the presence of influenza virus in placental tissue.

## 1. Introduction

Pregnant women are at increased risk of 2009 influenza A/H1N1 infection and its complications [1–3]. The viral infection does not only cause significant maternal morbidity, but also have a negative impact on pregnancy outcome in women hospitalized because of the infection. Preterm birth rates and stillbirth have been reported to be more prevalent in these women compared to noninfected women [4–7]. As well, it is reported that up to 25% of neonates born from mothers that recovered from severe influenza virus infection during pregnancy were small for gestational age [8]. It is unclear by which mechanism influenza virus infection causes these negative effects on pregnancy outcome. We hypothesized that chronic villitis, known to be associated with both intrauterine growth restriction and stillbirth and considered to be of viral origin in a considerable percentage of cases, may be responsible for these effects. We investigated whether chronic villitis was more prevalent in placentas of neonates whose mother had been affected by influenza infection during pregnancy compared to placentas of a cohort of uncomplicated pregnancies. We also investigated whether influenza could be demonstrated in placentas with chronic villitis.

## 2. Materials and Methods

We conducted a cohort study to investigate the effects of maternal influenza A/H1N1 infection on the course of pregnancy. The study protocol was approved by the local medical ethical review board and the clinical investigation was conducted according to the principles expressed in the Declaration of Helsinki. Data were collected from October 2009 until June 2010. All outpatients were asked to participate. Demographic data were collected from the charts of those who participated. Patients could be enrolled at any moment during pregnancy. At enrollment, patients filled out a questionnaire on vaccination against influenza virus, influenza like symptoms during the past six months, and, if this was the case, use of anti-influenza antiviral drugs. Influenza like symptoms were defined as fever ≥38,0°C, malaise, and cough eventually combined with myalgia. Patients were prospectively followed up. In case of influenza like symptoms, PCR on influenza on a swab taken from nose and pharynx was done. Data on pregnancy outcome were collected from the medical records.

Clinical cases of influenza virus infection were defined as influenza like symptoms: fever ≥38,0°C, malaise, and cough eventually combined with myalgia during the 2009 influenza pandemic in absence of a confirmatory PCR. Confirmed cases were defined as patients with a positive PCR on influenza. Placentas of women with influenza virus infection were examined histologically. If signs of chronic villitis were present, a PCR on placental tissue with chronic villitis was done.

DNA extraction of the paraffin embedded tissue was performed by conventional xylene deparaffinization, two xylene washes 60 minutes RT, two 96% ethanol washes, and one acetone wash followed by protease K digestion (18 hours, 56°C).

PCR was performed as follows. Nucleic acids were extracted using the total nucleic acid (for swabs) or DNA II tissue protocol (for placental tissue) with the MagNA pure LC nucleic acid isolation system (Roche Diagnostics, Basel, Switzerland). Each sample was eluted in 200 *μ*L buffer, which was sufficient for all real time PCR analyses. cDNA was synthesized by using MultiScribe reverse transcriptase (RT) and random hexamers (both from Applied Biosystems, Foster City, CA, USA). Each 200 *μ*L reaction mixture contained 80 *μ*L of eluted RNA, 20 *μ*L of 10X RT buffer, 5.5 mmol/L MgCl_2_, 500 *μ*mol/L of each deoxynucleoside triphosphate, 2.5 *μ*mol/L random hexamer, and 0.4 U of RNase inhibitor per microlitre (all from Applied Biosystems). After incubation for 10 minutes at 25°C, RT was carried out for 30 minutes at 48°C, followed by RT inactivation for 5 minutes at 95°C. The PCR consisted of a final concentration of 1X Universal Master Mix (Applied Biosystems), 900 nM of each primer, 150 nM of the influenza A probe, and 2 *µ*L of target cDNA and was made up to a volume of 25 *µ*L with nuclease free water (Promega Corp., Madison, USA). The real time quantitative PCR amplifications were measured in real time mode using the ABI7500 (Applied Biosystems).

Histologic examination of placentas was done by an experienced pathologist. The placentas were weighted without membranes and umbilical cord, and the weight was classified according to gestational age percentiles from Pinar et al. [9]. Routine hematoxylin and eosin stained slides from a minimum of 2 umbilical cord sections (at the fetal and placental side), a membrane roll, one sample from the umbilical cord insertion, and two slides of normal placental parenchyma, including decidua and chorionic plate, and additional slides from macroscopical abnormalities were reviewed. Chorioamnionitis was diagnosed based on the presence of polymorphonuclear cells (neutrophilic granulocytes) in the chorionic plate or the extraplacental membranes. Diagnosis of funisitis was based on the presence of neutrophilic granulocytes in the wall of the umbilical vein and/or arteries and Wharton's jelly. Chronic villitis was diagnosed as an infiltrate of lymphocytes and macrophages in the placental villi. In addition to the presence of chorioamnionitis, funisitis, or villitis, the severity of inflammation was graded mild, moderate, or severe, according to the staging and grading system of Redline [10] with slight modifications as previously published [11] and presented in Table 1.

### 2.1. Polymorphonuclear Cells (PMNs)

Placental parenchymal infarction was defined as an area of necrotic villi surrounded by an area of ischaemia with increased hyperchromasia of trophoblast and increased syncytial knotting. Fetal thrombotic lesions were scored as at least 5 avascular fibrotic villi without inflammation or mineralization or if adherent thrombi in stem vessels were present. An increase of nucleated red blood cells in the fetal circulation is the consequence of either anaemia or hypoxia and was scored when nucleated red blood cells were present in more than two capillaries in a random 10x field.

We compared the results of the histologic examination of the placentas with those of a historic cohort that was published before [11]. This cohort consisted of placentas derived from uncomplicated pregnancies with spontaneous onset of labor and vaginal delivery. The placentas in this cohort were examined in the same way and by the same laboratory and pathologist as the placentas collected in this study.

## 3. Results

In total 145 patients participated in our study. Data were incomplete in nine patients. Of the participants, 29 (20%) were clinical cases of influenza virus infection, of which 6 were tested by PCR. All PCRs tested positive. All 29 pregnant women gave live birth and both mothers and children were discharged in good health. We were able to collect 15 out of 29 placentas.

Of these 15, 10 were clinical cases and 5 were PCR proven influenza cases. Baseline characteristics and pregnancy outcome of the 15 patients are presented in Table 2.

Gestational age at onset of symptoms varied from 2 to 32 weeks with a median of 21 weeks of gestation. There were 5 patients with infection in the first, 8 in the second, and 2 in the third trimester of pregnancy. None of the patients had to be admitted to the hospital. Three of the patients were treated with oseltamivir. Other treatments included amoxicillin (±clavulanic acid) in 4 patients, paracetamol in 1 patient, and no treatment in 4 patients. In all but one patient, symptoms subsided within 10 days.

In 7 out of 15 placentas (47%) chronic villitis was present. Results are shown in Table 3. All 7 PCRs run on placental tissue were negative.

When comparing characteristic between those patients whose placentas showed villitis to those whose placentas did not, there were no differences in average duration from infection to delivery (153 days (SD 64 days) and 140 days (SD 67 days) for cases with resp. without chronic villitis). There were also no differences in placental weight, birth weight of the neonates and number of neonates that were small for gestational age. In the placentas with chronic villitis 3 out of 7 placentas were below 10th percentile for placental weight according to gestational age, while all placentas without chronic villitis were of normal weight. Results are shown in Table 4. There were no differences in chorioamnionitis, funisitis, infarction, or thrombosis (results not shown).

## 4. Discussion

In our study sample, chronic villitis was present in 47% of placentas. This appears to be considerably higher than the 24% in our historic cohort and even more different from the 5–15% reported by Redline [10]. Comparing the 47% in our cohort with the 24% in our historic cohort yields an absolute difference of 23% with a 95% confidence interval for difference of −3 to 49%. Interestingly, the placentas of all three women treated with oseltamivir demonstrated chronic villitis (all grade 1). However, the relatively small number of placentas that we were able to study makes it hard to draw any firm conclusions on these numbers.

The etiology of chronic villitis is not fully elucidated yet. Some cases can be attributed to infections with microorganisms like cytomegalovirus or *Treponema pallidum*. A large proportion of cases of chronic villitis however were classified as villitis of unknown etiology (VUE) and may reflect a noninfectious immune response. Which proportion of chronic villitis has to be attributed to viral infections and which proportion has to be classified as VUE is still a matter of debate [10, 13]. In some cases these conditions can be distinguished by using certain characteristics on routine histological examination [10]. However, it is also reported that 41% of lesions originally classified as VUE appear to have a viral origin when examined by electron microscopy [14]. Regardless of its etiology, recent reports including a meta-analysis have confirmed the association of VUE with intrauterine growth restriction and, to a lesser extent, with pregnancy loss [15, 16].

In our series we found five grade 1 and two grade 2 lesions. The small size of our study sample limits the ability to detect differences in pregnancy outcomes between cases with and without chronic villitis. We found no significant difference in birth weight of neonates and there were also no significant differences between placental weight and number of placentas with placental weight < p10, although the latter almost reached statistical difference (*P* = 0, 07).

We were not able to demonstrate the presence of 2009 influenza A/H1N1 in placental tissue with histological signs of chronic villitis. Several reasons might exist for this result. First, chronic villitis may be unrelated to influenza infection. Second, the pregnant women in our cohort were relatively mildly affected by the virus. Pregnant women who are more severely affected and have viraemia may be more prone to placental infection by influenza [17]. Nevertheless, for seasonal influenza A H1N1 placental infection and vertical transmission have been reported even when maternal symptoms were mild [18]. Third, the virus might also have been cleared from placental tissue since the time between infection and delivery was relatively long in our cohort. Fourth, we may not have selected the appropriate patients and/or placentas to examine, since 10 out of 15 of our cases were clinical cases without a confirmative PCR. However, given that all patients tested by PCR on pharyngeal swab had a proven infection, a large proportion of patients that were not tested but had similar symptoms during the influenza pandemic will probably have had influenza infection as well. Therefore, we expect a large proportion of clinical cases to be actual influenza infections. Finally, placental tissue was embedded in paraffin. This may have decreased the sensitivity of the PCRs, since both natural degradation of RNA and the fixation and paraffin-embedding procedure may interfere with the transcription and amplification of RNA [19]. On the other hand, we selected those pieces of placental tissue that showed chronic villitis, hypothesizing that we would have optimal chances of finding evidence of placental infection by influenza if present.

Our findings are in line with results from Turkey [20]. In a series of seven placentas derived from pregnancies complicated by proven pandemic influenza A H1N1 infection, Kanmaz et al. performed PCR on random pieces of placental tissue not related to any histologic abnormalities. Like us, they did not find evidence of viral replication in placental tissue.

Still, influenza infection may be related to chronic villitis. The increased prevalence of chronic villitis of unknown etiology in women with autoimmune diseases and in ovum donor pregnancies suggests that it results from a disturbance of normal pregnancy tolerance, which is predominantly regulated by several types of T cells [16]. The immunogenic activity of these cells, however, is influenced by cytokines that are known to be increased in influenza virus infection [21, 22]. We therefore hypothesize that the burst of inflammatory cytokines accompanying influenza virus infection may produce lesions of chronic villitis. Given the limitations of our study and the limited literature on this subject further studies are needed to affirm or deny this hypothesis.

## 5. Conclusions

In our small series, we found a high proportion of placentas with chronic villitis in women infected with 2009 influenza A/H1N1 during pregnancy. We could not demonstrate that chronic villitis was caused by direct infection of the placenta with influenza virus. Our results underline the need for further investigation of whether the immune response triggered by influenza virus infection is responsible for the increased rates of serious adverse events that occur in pregnancies complicated by influenza virus infection.

## Figures and Tables

**Table 1:** Histological classification of chorioamnionitis, funisitis, and villitis.

Grade	Chorioamnionitis	Funisitis	Villitis
0	No inflammation	No inflammation	No inflammation
0.5	Sporadic PMN in chorionic plate/membranes		
1	Frequent PMN in chorionic plate/membranes	Inflammation present in the wall of 1 vessel (vein)	1 section with 1 focus of chronic inflammation of >5 villi
2	Invasion of chorionic plate, large infiltrate in chorionic plate/membranes	Inflammation present in the wall of 2 vessels (vein and artery)	2-3 sections with each having 1 focus of chronic inflammation of >5 villi
3	Same as 2, including microabscesses in chorionic plate	Inflammation present in the wall of 3 vessels (vein and 2 arteries)	3 sections with each having >2 foci of chronic inflammation of >5 villi

PMNs: polymorphonuclear cells.

**Table 2:** Baseline characteristics and pregnancy outcome of pregnant women with 2009 influenza A/H1N1 infection during pregnancy whose placentas were examined.

Number of patients (*n*)	15
Age (y)	
Mean (SD)	33 (5,8)
Parity	
Nulliparous (*n* (%))	5 (33)
Parous (*n* (%))	10 (67)
Comorbidity	
None (*n* (%))	11 (73)
Asthma (*n* (%))	1 (7)
Hypertension (*n* (%))	1 (7)
Other systemic diseases* (*n* (%))	2 (13)
Mode of delivery	
Vaginal delivery (*n* (%))	10 (67)
Cesarean section (*n* (%))	5 (33)
Gestational age	
Mean (days (SD))	280 (10)
Preterm <37 weeks (*n* (%))	1 (7)
Preterm <34 weeks (*n* (%))	0 (0)

*One patient had rheumatoid arthritis and one patient had polycystic ovary syndrome.

**Table 3:** Number of placentas with chronic villitis, divided by proven or suspected infection and grade of villitis.

	Villitis of any grade (*n*, (%))	Villitis of grade 1 (*n*)	Villitis of grade 2 (*n*)	Villitis of grade 3 (*n*)
PCR confirmed cases (*n* = 5)	3 (60)	3	0	0
Clinical cases (*n* = 10)	4 (40)	2	2	0
Total (*n* = 15)	7 (47)	5	2	0
Historic cohort* (*n* = 271)	65 (24)	46	16	3

*Historic cohort consists of placentas of women with uncomplicated pregnancy and vaginal delivery, as described in Section 2 [11].

**Table 4:** Placental weight, number of placentas with weight < p10, birth weight, and number of neonates with birth weight < p10 divided by the absence or presence of villitis.

	Placental weight (grams, mean (SD))	Placental weight < p10 (*n*, (%))	Birth weight (grams, mean (SD))	Birth weight < p10 (*n*, (%))
No villitis (*n* = 7)	487 (93)	0 (0)	3317 (510)	1 (14)
Chronic villitis (*n* = 8)	447 (57)	3 (38)	3176 (480)	0 (0)

Placental weight for gestational age was classified according to Pinar et al. [9].

Birth weight for gestational age was classified according to the new Dutch reference curves for birth weight by gestational age [12].
